# The effect of pre-pregnancy body mass index and excessive gestational weight gain on the risk of gestational diabetes in advanced maternal age

**DOI:** 10.18632/oncotarget.17651

**Published:** 2017-05-07

**Authors:** Beibei Dong, Hong Yu, Qiong Wei, Mengmeng Zhi, Chunhua Wu, Xiangyun Zhu, Ling Li

**Affiliations:** ^1^ Department of Endocrinology, ZhongDa Hospital of Southeast University, Nanjing, 210009, PR China; ^2^ Department of Gynecology and Obstetrics, ZhongDa Hospital of Southeast University, Nanjing, 210009, PR China; ^3^ School of Medicine, Southeast University, Nanjing, 210009, PR China

**Keywords:** pre-pregnancy body mass index, excessive gestational weight gain, gestational diabetes, advanced maternal age

## Abstract

**Background and purpose:**

With the popularization of a two-child policy in China, the number of pregnant women of advanced maternal age will increase steadily. We aimed to assess the association between pre-pregnancy body mass index (BMI) and weight gain in the first and second trimester and the risk of gestational diabetes (GDM) in the advanced maternal age group and control group defined as maternal age of 20–35 years.

**Results:**

The risk of GDM for obesity before pregnancy was 2.707 (95% CI: 1.042–7.029) folds and 3.612 (95% CI: 1.182–11.039) folds in the control group and advanced maternal age group, respectively. Excessive weight gain in the first trimester was significant related to a higher risk of developing GDM with the odds ratio (OR) of 2.655 (95% CI: 1.265–5.571) and 4.170 (95% CI: 1.437–12.100) in the control group and advanced maternal age group, respectively.

**Materials and methods:**

This prospective cohort study included 565 pregnant women with singleton pregnancy who were recruited in their first prenatal visit from the antenatal clinic in March and December 2016. Maternal weight was recorded before pregnancy, in the first prenatal visit and at the time of screening oral glucose tolerance test (OGTT). All women underwent 2 h 75g-OGTT at 24–28 weeks (24 weeks on average). GDM was diagnosed according to the standards issued by the Ministry of Health of China in 2011.

**Conclusions:**

Elevated pre-pregnancy BMI independently increases the risk of GDM, particularly in advanced maternal age. Excessive weight gain in the first trimester is significantly associated with the incidence of GDM regardless of pre-pregnancy BMI.

## INTRODUCTION

In October 2015, China announced that the traditional one-child policy finally had been replaced by a universal two-child policy. Compared the traditional one-child policy, universal two-child policy will increase the population [1]. Besides, with the development of social industrialization, more women are choosing to delay childbearing until their education is completed or their professional careers are established [2]. Consequently, the number of pregnant women in advanced maternal age, usually defined as pregnancy at the age of 35 years and over, have increased steadily [3].

For decades, it has been recognized that women of advanced maternal age have a higher chance of developing impaired glucose tolerance (IGT) and gestational diabetes (GDM) [4, 5]. Moreover, contemporary childbearing women with advanced maternal age were more likely to be multiparous, college-educated and obese before pregnancy [3]. Several studies have shown that maternal obesity has a known association with poor maternal and fetal outcomes including GDM [6, 7], particularly in advanced maternal age [8], which is already a risk factor of GDM [9]. Large numbers of studies have clarified that the relationship between the obesity before pregnancy and the risk of GDM in common pregnant women aged < 35 years [10–12]. Furthermore, several studies suggested that excessive weight gain during the pregnancy is associated with GDM [13–15]. Whereas there are few studies to demonstrate the correlation between obesity before pregnancy and excessive weight gain during the pregnancy and the risk of GDM in advanced maternal age [3].

This study was designed to compare the association between the pre-pregnancy BMI and weight gain in the first and second trimester and the risk of GDM in the advanced maternal age group (aged 35 years and over) and the control group (aged 20–35 years).

## RESULTS

### Baseline characteristics of study population prior to pregnancy (Table 1)

Five hundred sixty-five pregnant women were included in the study, 301 of whom were included in the control group and 274 of whom were included in the advanced maternal age group. The characteristics of women with advanced maternal age and those in the control group stratified based on maternal age are listed in the Table 1. Women with advanced maternal age were more likely to be multiparous and overweight or obese before pregnancy compared with women in the control group. Education, maternal height and age at menarche were not significantly different between the control and advanced maternal age group. Family history of hypertension and diabetes did not affect the risk of gestational diabetes (GDM) in two groups of the study.

**Table 1:** Baseline characteristics of the study population according to maternal age

Baseline characteristics	Entire cohort N = 565	Age grouped		
		Control group (*N* = 361)	Advanced maternal age group (*N* = 204)	*P*-value
**Maternal age (yeas)**	31 (27–35)	28 (26–30)	36 (35–38)	< 0.001^c^*
**Education (years)**	15.02 ± 2.40	15.11 ± 2.16	14.86 ± 2.77	0.227^d^
**≤ 12^a^**	107 (18.90)	57 (15.80)	50 (24.50)	
**12–16^b^**	414 (73.30)	281 (77.80)	133 (65.20)	
**> 16**	44 (7.80)	23 (6.40)	21 (10.30)	
**Maternal height (cm)**	161.94 ± 4.62	162.07 ± 4.58	161.72 ± 4.70	0.559^d^
**Pre–gestational weight (kg)**	56.00 (51.50–62.00)	55.00 (50.50–62.00)	58.00 (53.00–63.00)	0.005^c^*
**Pre–gestational BMI (kg/m^2^)**	21.48 (19.66–23.62)	20.94 (19.29–23.44)	22.05 (20.43–23.89)	< 0.001^c^*
**–18.5**	65 (11.50)	54 (15.00)	11 (5.40)	
**18.5–23**	328 (58.10)	205 (56.80)	123 (60.30)	
**23–27.5**	135 (23.90)	80 (22.20)	55 (27.00)	
**27.5–**	37 (6.50)	22 (6.00)	15 (7.30)	
**Age at menarche (years)**	13.73 ± 1.28	13.71 ± 1.25	13.77 ± 1.34	0.878^d^
**Parity (numbers)**	2.12 ± 1.18	1.83 ± 1.08	2.63 ± 1.19	< 0.001^d^*
**≥ 2 (*n*** **(%))**	287 (50.80)	112 (31.02)	175 (85.78)	
**Abortion**	0.82 ± 1.05	0.63 ± 0.95	1.15 ± 1.14	< 0.001^d^*
**Family history of hypertension (*n*** **(%))**	55 (9.73)	38 (10.53)	17 (8.33)	0.399^e^
**Family history of diabetes (*n*** **(%))**	32 (5.66)	23 (6.37)	9 (4.41)	0.334^e^

Data are presented as *n* (%), mean ± SD, or median (interquartile range) as appropriate.

* Statically significant *P*-value < 0.05.

^a^: Accepted education for 12 years means already obtained the degree of high school.

^b^: Accepted education for 16 years means already obtained the degree of bachelor.

^c^: Mann-Whitney *U* test for comparison of skew distribution quantitative variables between the control group and the advanced maternal age group.

^d^: Student's *t* test for comparison of normally distributed quantitative variables between the control group and the advanced maternal age group.

^e^: *χ*^2^ test and Fisher's Exact Test for comparison of qualitative variables between the control group and the advanced maternal age group.

Abbreviation: BMI: body mass index.

### The effect of BMI before pregnancy on the incidence risk of GDM (Table 2)

**Table 2:** The effect of BMI before pregnancy on the incidence of gestational diabetes (GDM) between the control group and the advanced maternal age group

	Control group *N* = 361 (24.93%)^a^			Advanced maternal age group *N* = 204 (36.27%)^b^		
	*n* (%)^c^	OR	Adjusted OR	*n* (%)^d^	OR	Adjusted OR
Pre-BMI (kg/m^2)^						
–18.5	10 (18.52)	0.786 (0.367–1.681)	0.762 (0.348–1.667)	2 (18.18)	0.497 (0.102–2.411)	0.59 (0.119–2.921)
18.5–23	46 (22.44)	1	1	38 (30.89)	1	1
23–27.5	23 (28.75)	1.395 (0.777–2.503)	1.344 (0.739–2.444)	25 (45.45)	1.864 (0.969–3.586)	1.919 (0.984–3.74)
27.5–	11 (50.00)	3.457 (1.408–8.483)*	2.707 (1.042–7.029)*	9 (60.00)	3.355 (1.115–10.095)*	3.612 (1.182–11.039)*

Data are presented as *n* (%).

a and b: N means the number of women in the control group and advanced maternal age group, respectively; % means the percentage of women diagnosed with GDM in the total numbers of women in the two groups, respectively.

c and d: n means the number of women diagnosed with GDM; % means the percentage of women diagnosed with GDM in a subgroup of BMI categories.

* Statically significant *P*-value < 0.05.

Abbreviations: Pre-BMI: pre-pregnancy body mass index; OR: odds ratio; CI: confidence interval.

Adjusted for education; age at menarche; parity; abortion and family history of diabetes.

Data extracted from this sample showed that body mass index (BMI) before pregnancy stratified based on the criteria for Asian population was significantly related to the risk of GDM. According to the criteria [16], BMI was categorized as the underweight (< 18.5 kg/m^2^), normal weight (18.5–23 kg/m^2^), overweight (23–27.5 kg/m^2^) and obesity (≥ 27.5 kg/m^2^) in the prior to pregnancy.

The proportion of GDM was 36.27% in the advanced maternal age group compared to 24.93% in the control group (*p* value = 0.004). Similarly, the incidence of GDM increased with increasing pre-pregnancy BMI in both the advanced maternal age group and the control group. The prevalence of GDM among pre-pregnancy BMI categories of underweight, normal weight, overweight and obesity was 18.18%, 30.89%, 45.45% and 60.00% respectively in the advanced maternal age group compared to 18.52%, 22.44%, 28.75% and 50.00% in corresponding BMI categories of the control group, respectively. (*P* value = 0.090, 0.001, 0.399, 0.035). The risk of GDM for obesity were 2.707 (95% confident interval (CI): 1.042–7.029) folds and 3.612 (95% CI: 1.182–11.039) folds in the control group and advanced maternal age group, respectively.

### Further assessment of gestational weight gain in the first and second trimester on the risk of GDM according to pre-pregnancy BMI categories (Table 3)

**Table 3:** The impact of gestational weight gain during the first and second trimesters on the risk of GDM according to the category of pre-pregnancy BMI

	Control group *N* = 361 (24.93%)			Advanced maternal age group *N* = 204 (36.27%)		
	n (%)^a^	OR	Adjusted OR	*n* (%)^b^	OR	Adjusted OR
WG 1st						
Inadequate	41 (25.79)	1.969(0.969–4.001)	2.048 (0.992–4.232)	20 (32.79)	2.114 (0.750–5.958)	2.721 (0.868–8.533)
Normal	12 (15.00)	1	1	6 (18.75)	1	1
Excessive	37 (30.33)	2.467 (1.195–5.093)*	2.655 (1.265–5.571)*	48 (43.24)	3.302 (1.259–8.656)*	4.170 (1.437–12.100)*
WG 2nd						
Underweight	10 (18.52)	1.034 (0.766–1.396)	1.023 (0.732–1.430)	2 (18.18)	0.318 (0.037–2.755)	0.301 (0–0)
Non –excessive	9 (20.00)	1	1	2 (22.22)	1	1
excessive	1 (11.11)	0.500 (0.055–4.528)	0.635 (0.065–6.155)	2 (1)	0	0.046 (0–0)
Normal weight	46 (22.43)	0.980 (0.873–1.099)	0.968 (0.862–1.087)	38 (30.89)	0.960 (0.834–1.106)	0.939 (0.808–1.092)
Non –excessive	30 (24.00)	1	1	30 (33.33)	1	1
excessive	16 (20.00)	0.792 (0.399–1.570)	0.714 (0.348–1.464)	8 (24.24)	0.640 (0.258–1.588)	0.594 (0.224–1.576)
Overweight	23 (28.75)	1.173 (0.987–1.395)	1.199 (0.996–1.444)	25 (45.45)	0.899 (0.759–1.064)	0.822 (0.666–1.014)
Non –excessive	5 (16.66)	1	1	13 (54.17)	1	1
excessive	18 (36.00)	2.812 (0.917–8.624)	3.436 (1.015–11.633)*	12 (38.71)	0.534 (0.181–1.574)	0.479 (0.144–1.599)
Obesity	11 (50.00)	0.923 (0.663–1.284)	1.372 (0.646–2.915)	9 (60.00)	1.092 (0.781–1.527)	5.470 (0–)
Non –excessive	6 (60.00)	1	1	6 (66.67)	1	1
excessive	5 (41.67)	0.476 (0.086 –2.628)	0.032 (0–5.106)	3 (50.00)	0.500 (0.060–4.153)	0.767 (0.026–22.784)

Data are presented as *n* (%).

a and b: n means numbers of women diagnosed with GDM; % means the percentage of women diagnosed with GDM in total population of the corresponding subgroup.

*statically significant *P*-value < 0.05.

Adjusted for education; age at menarche; parity; abortion; family history of diabetes.

WG 1ST: weight gain in the first trimester from the last menstrual to the first prenatal visit according to IOM (2009) recommendation for 0.5–2 kg;

Inadequate: weight gain < 0.5 kg; Adequate: weight gain for 0.5–2 kg; Excessive: weight gain ≥ 2 kg;

WG 2nd: weight gain in the second trimester from the first prenatal visit to the time of OGTT screening tests according to IOM (2009) guidelines based on BMI categories. Non-excessive: < recommended weight gains. Excessive: ≥ recommended weight gains.ww

The gestational weight gain during the first and second trimester was categorized according to the Institute of Medicine (IOM) modified criteria (2009). In the first trimester, gestational weight gain 0.5–2 kg was recommended by the IOM modified criteria. According to those, pregnant women of two groups were stratified into inadequate weight gain, normal weight gain and excessive weight gain.

In the first trimester, the incidence of GDM in women with inadequate weight gain and excessive weight gain were 25.79% and 30.33% in the control group compared to 32.79% and 43.24% in the advanced maternal age group, respectively. (*P* value = 0.299, 0.001) The effect of women with excessive weight gain on the risk of GDM was 2.655 (95% CI: 1.265–5.571) folds and 4.170 (95% CI: 1.437–12.100) folds in the control and advanced maternal age group. In the second trimester, women with underweight, normal weight, overweight and obesity are recommended to gain different amount of gestational weight according to the IOM modified criteria. Thus, pregnancy weight gains during the second trimester were classified as non-excessive and excessive weight gain on the basis of it. The risk of GDM for overweight with excessive weight gain was 3.436 (95% CI: 1.015–11.633) folds in the control group. Weight gains were not significantly associated with the risk of GDM in the underweight, normal weight and obesity of the two groups.

## DISCUSSION

This study is a first step in demonstrating the significant association between BMI before pregnancy and weight gain during the first and second trimester and the risk of GDM in advanced maternal age. Overall, the prevalence of GDM gradually increases along with the rising maternal age. We found that BMI of obese women before pregnancy in the control group and advanced maternal age group was significantly associated with the incidence of GDM, particularly in the advanced maternal age group. Excessive weight gain during the first trimester was related more significantly to the risk of GDM than weight gain in the second trimester, and this was more striking in the advanced maternal age group than in the control group.

Our study showed that obese women before pregnancy in the control group and advanced maternal age group were 2.707 (95%CI: 1.042–7.029) times and 3.612 (95%CI: 1.182–11.039) times more likely to develop GDM as compared to normal weight women. The association between pre-pregnancy BMI and the incidence risk of GDM was similar to those found in other studies [11, 19]. Recently, a population-based cohort study has demonstrated that pre-pregnancy obesity was associated with higher risk of GDM after adjustment for a range of factors including age, area of residence, highest education completed, parity and lifestyle factors and obese women before pregnancy were 2.68 (95% CI: 1.98–3.62) times as likely to develop GDM as compared to women with normal weight [12]. However, those findings only identified the association between obesity before pregnancy and the risk of GDM in common maternal age. Our novel finding was that in the advanced maternal age, obesity before pregnancy was 3.612 (95% CI: 1.182–11.039) times more likely to develop GDM as compared to normal weight women. This risk of developing GDM in advanced maternal age group was significantly higher than the risk in the control group. These obese women before pregnancy have decreased insulin sensitivity as compared with lean or average weight women [19, 21] and the underlying metabolic defects related to the develop GDM are decreased insulin sensitivity coupled with inadequate insulin supplementation [10].

In the current study, excessive weight gain during the first trimester was significantly related to GDM with the odds ratio (OR) of 2.655 (95% CI: 1.265–5.571) and 4.170 (95%CI: 1.437–12.100) in the control group and advanced maternal age group, respectively. Similarly, Hedderson et al [14] demonstrated that women with excessive early pregnancy (12 weeks of gestation on average) weight gain may increase the risk of GDM. In another study, early rate of gestational weight gain (RGWG) was significantly associated with high risk of developing GDM [15]. We also observed that weight gain in the second trimester was not significantly association with GDM. In compliance with a retrospective study of 2789 women, the study demonstrated that rate of gestational weight gain (RGWG) during the mid-pregnancy was not significantly associated with any adverse pregnancy outcomes including GDM [15]. It's possible that gestational weight gain in the first trimester consists of more maternal body fat and is associated with changes maternal metabolism and decreased insulin sensitivity, and these changes provide a large amount of substrate to meet fetoplacental needs during pregnancy [22]. Whereas weight gain in the second and later trimester mainly consists of increased fetoplacental proportions. Thus, large gain of maternal fat mass in the first trimester could have a significant effect on succeeding insulin resistance compared to later gains in lean mass or fetal mass [14, 23, 24]. However, those findings were mainly focused on the common pregnant women. Our study investigated the strongly association between excessive weight gain in the first trimester and the risk of GDM in advanced maternal age. This finding has important clinical and healthy implication as pregnant women in advanced maternal age with excessive weight gain in the first trimester were 4.170 folds more likely to develop GDM regardless of pre-pregnancy BMI. Thus, lifestyle intervention to control excessive weight gain may be an effective strategy to decrease the incidence of GDM in advanced maternal age, which predispose to GDM.

A distinct significance of this study was the first to compare and analyze the relationship in the control group and advanced maternal age group between the values of BMI before pregnancy and excessive gestational weight gain and the incidence risk of GDM. Several studies had demonstrated the relationship between pre-pregnancy BMI and gestational weight gain in the first and second trimester and the risk of GDM in general population [12, 14, 19, and 22]. However, there are few studies discussing the association between pre-pregnancy BMI and weight gain during pregnancy in advanced maternal age, a noteworthy population. In addition, the present study design along with enrollment also minimize the risk of selection bias.

Despite our study's strengths, there were still some limitations. One limitation of this study was that the study population were recruited from a single hospital affiliated to university, therefore, the study population may not be representation of the entire population. Additionally, the study used self-reported pre-pregnancy weight. It previously has been shown that self-reported pre-pregnancy weight was well associated with actual weight [14, 25]. Another limitation was that we did not record information regarding diet and physical activity during pregnancy, thus were not able to assess their effects on gestational weight gain and GDM.

The study was the first to evaluate and identify the significant association in advanced maternal age women between pre-pregnancy BMI and gestational excessive weight gain in the first trimester and the incidence risk of GDM, which may bring heavy healthcare burden for families and society. More studies of large samples are needed to evaluate the effects of excessive weight gain during the different gestational trimesters on pregnancy outcomes in advanced maternal age. Additionally, further interventional studies in advanced maternal age are required to assess the efficacy of limiting excessive gestational weight gain, especially in the first trimester through diet and exercise, on the risk of GDM.

In conclusion, our study is the first to demonstrate the significantly association between pre-pregnancy BMI and weight gain in the first trimester and the risk of GDM in an advanced maternal age population compared to a control group. However, this relationship is based on the context of this study, any casual interpretation of the relationship is not accurate and limited. Further well designed and international studies are required to determine whether the significant association between BMI before pregnancy and weight gain during the specific trimester in advanced maternal age from this study are generalizable to all advanced maternal age population. Given the long-term health implication of GDM on the effect of maternal and fetal health, this study also suggests that we should pay more attention to the advanced maternal age to prevent a heavy health burden for families and for society. Thus, identifying potential strategies for prevention of early excessive weight gain, especially during the first trimester, may have far-reaching benefits. Obstetricians should provide more guidance in monitoring and controlling gestational weight gain, particularly in early pregnancy, and more intervention studies are required to verify whether changes in gestational weight gain in advanced maternal age will lead to a decrease in the incidence of gestational diabetes.

## MATERIALS AND METHODS

### Study design and population

This prospective cohort study of 565 women with singleton pregnancy was conducted at the ZhongDa Hospital affiliated to Southeast University, Nanjing in China. The study was approved by the hospital's human research ethnics committee and adhered to the STROBE guidelines for observational studies. Written informed consent was obtained from all pregnant women.

The pregnant women were recruited by consecutively in the first prenatal visit from the antenatal clinic between March and December 2016. Pregnant women were eligible for the study if they were 20 to 55 years of age with singleton pregnancy. Exclusion criteria included maternal age less than 20 years and more than 55 years; pre-gestational diabetes; autoimmune diseases prior to pregnancy, pre-pregnancy cardiovascular diseases and severe hepatic and renal disorders before pregnancy. According to the cut-off points of advanced maternal age which was recommended as 35 years of age, the enrolled women were divided into the control group and the advanced maternal age group, respectively.

We collected information on demography, maternal age, education and employment, pre-gestational weight and height, last menstruation, pregnancy and obstetrical history, history of GDM, hypertension, and macrosomia in previous pregnancies and family history of DM. Self-reported pre-pregnancy weight and height measured without shoes was gathered at the first clinical visit by asking the weight of the last menstrual period or average weight over the past three months and the weight at the first clinical visit and when the 75g OGTT were measured with light clothes in the antenatal clinic. Maternal BMI (kg/m^2^) of pre-pregnancy, the first clinical visit and the period of proceeding 75g OGTT were calculated by weight in kilogram (kg) divided by the square of height in meters (m). Specific BMI classifications were determined on the basis of the appropriate BMI cut-off points for overweight and obesity related to public health action in Asian population. For many Asian population, additional trigger points for public health action were identified as 23 kg/m^2^ or higher, representing increased risk, and 27.5 kg/m^2^ or higher as representing high risk. The suggested categories are as follows: less than 18.5 kg/m^2^ underweight; 18.5–23 kg/m^2^ increasing but acceptable risk; 23–27.5 kg/m^2^ increased risk; and 27.5 kg/m^2^ or higher high risk [16].

Weight gain during the first trimester from pre-pregnancy to the first prenatal visit and the second trimester from the first prenatal visit to the time of carrying out 75g OGTT were classified as inadequate weight gain, normal weight gain and excessive weight gain according to the Institute of Medicine (IOM) modified criteria (2009) for weight gain during pregnancy [17]. In the first trimester, gestational weight gain 0.5–2 kg was recommended by the IOM (2009) which was applicable to all pregnant women regardless of pre-pregnancy BMI. The modified IOM criteria are formulated as a range for each category of pre-pregnancy BMI based on the World Health Organization (WHO) cut-off points for the BMI categories and estimated an appropriate range of weight gains during the second trimester. According the maternal pre-pregnancy BMI, the women of underweight, normal weight, overweight and obesity were recommended to gain weight 0.51 kg/week, 0.42 kg/week, 0.28 kg/week and 0.22 kg/week in the second trimester, respectively. However, one of the limitations of the guideline is that these recommendations are not intended for use in areas of the world where women are substantially shorter or thinner than American women. Thus, we selected the appropriate and recommended criteria for Asian population related to public health action. In the light of IOM (2009), we calculated the recommended weight gain in the second trimester as the weeks from the first clinical visit to the time of screening 75 g OGTT multiplied by the recommended rate of weight gain based on the different pre-pregnancy BMIs. Non-excessive and excessive weight gain mean that weight gain is less than and more than the recommended weight gain, respectively.

### Diagnostic criteria of GDM

At 24–28 weeks, maternal plasma glucose concentrations were measured by 1h and 2h after a 75g oral glucose tolerance test (OGTT). Gestational diabetes (GDM) was diagnosed according to the standards issued by the Ministry of Health of China in 2011, which was on the basis of International Association of Diabetic Pregnancy Study Group (IADPSG) guidelines [18]. Briefly, GDM is defined as having one or more abnormal values from 2-hour 75-g OGTT between 24 and 28 weeks of gestation, with the cut-off points of 5.1 mmol/L (92 mg/dl) for fasting, 10.0 mmol/L (180 mg/dl) for 1 hour, and 8.5 mmol/L (153 mg/dl) for 2 hours. Women diagnosed with GDM subsequently received both diet and/or insulin treatment at hospital, which was the basis of selection for the period of gestational weight gain until the diagnosis of GDM.

### Statistical analysis

All statistical analysis was conducted using SPSS version 16.0 statistical software package. Continuous variables were descripted as mean ± standard deviation if the continuous variables were followed by normal distribution. If the distribute of continuous variables were skewed, then they were descripted as median and interquartile range. Categorical variables were reported as count and percentage. Interdepend T tests and Mann Whitney U tests were conducted in continuous variables, while Pearson Chi-Square and Fisher's Exact Test were applied to categorical variables. To evaluate whether BMI before pregnancy and gestational weight gain in the first and second trimester were associated with the incidence risk of GDM in the control group and the advanced maternal age group, binary logistic regression models were built up with enter variable selection (probability for removal > 0.1) adjusting education, age at menarche, parity, abortion, and family history of diabetes. Results from those analyses were presented as odds ratio (OR) with 95% confidence interval (CI). All tests were two-sided and *p* < 0.05 was considered as statistically significant.

## Abbreviations

BMI: body mass index
GDM: gestational diabetes
OR: odds ratio
CI: confidence interval
OGTT: oral glucose tolerance test
IGT: impaired glucose tolerance
IOM: Institute of Medicine
RGWG: rate of gestational weight gain
WHO: World Health Organization
IADPSG: International Association of Diabetic Pregnancy Study Group
